# Energy Efficient Resource Allocation for M2M Devices in 5G

**DOI:** 10.3390/s19081830

**Published:** 2019-04-17

**Authors:** Anum Ali, Ghalib A. Shah, Junaid Arshad

**Affiliations:** 1 Department of Computer Science and Engineering, University of Engineering and Technology, Lahore 54890, Pakistan; 2 Sultan Quboos IT Chair, University of Engineering and Technology, Lahore 54890, Pakistan; ghalib@kics.edu.pk; 3 Department of Computer Science and Engineering, University of Engineering and Technology, Lahore 54890, Pakistan; junaidarshad@uet.edu.pk

**Keywords:** machine-to-machine, energy efficiency, resource allocation, 5G

## Abstract

Resource allocation for machine-type communication (MTC) devices is one of the keys challenges in the 5G network as it affects the lifetime of battery powered devices and also the quality of service of the applications. MTC devices are battery restrained and cannot afford a lot of power consumption due to spectrum usage. In this paper, we propose a novel resource allocation algorithm termed threshold controlled access (TCA) protocol. We propose a novel technique of uplink resource allocation in which the devices make a decision of resource allocation blocks based on their battery status and related application’s power profile that eventually leads to required quality of service (QoS) metric. The first phase of the TCA algorithm selects the number of carriers to be allocated to a certain device for the better lifetime of low power MTC devices. In the second phase, the efficient solution is implemented through inducing a threshold value. A certain value of the threshold is selected through a mapping based on a QoS metric. The threshold enhances the selection of subcarriers for less powered devices, such as small e-health sensors. The algorithm is simulated for the physical layer of the 5G network. Simulation results show that the proposed algorithm is less complex and achieves better performance when compared to existing solutions in the literature.

## 1. Introduction

Machine-to-machine (M2M) communication network is the base technology for enabling Internet of Things (IoT) [1]. It establishes the concept of autonomous data transfer that can be used in numerous applications, such as smart grids, enterprise, e-health, telematics, and security, etc. For now, this network is based on a cellular network but aims to evolve towards the fifth generation mobile communication system (5G). The 5G network is in an experimental phase; therefore, there are many challenges for its deployment and efficient functionality. Some of the challenges are small burst traffic, time controlled functions, varied power requirements, massive transmissions, high mobility, a large number of devices, and different QoS demands, etc. One of the most critical requirements is energy efficiency since the machine-type communication (MTC) [2] devices run on small batteries that are difficult to recharge or replace; therefore, they cannot afford complex computations that are usually used during radio resource management [3,4]. The energy efficiency of the devices should be increased by a factor of at least 10 so that the lifetime of batteries is increased tenfold. The 5G deployment meets several requirements of MTC applications, such as increasing reliability and scalability, ensuring availability, reducing latency and providing interoperability; moreover, it provides functionalities to boost energy efficiency and updates security [5]. The key enabling technologies provided by 5G are advance coding techniques, congestion reduction techniques, supporting heterogeneous networks, and enabling software defined networks (SDN) and network function virtualization (NFV) to make core and backhaul networks flexible. New waveforms are also investigated in 5G; previously, orthogonal frequency division multiplexing (OFDM) has gained much of the popularity but 5G offers new modulation techniques including a filter bank multicarrier (FBMC) scheme. FBMC provides high spectrum sensing resolution, whereas spectrum sensing performance is degraded in the OFDM technique. Efficient radio resource allocation strategies could play a major role in the performance of FBMC wireless networks. Many research efforts in this area are related to downlink resource allocation, which has restricted the effectiveness of the energy efficiency of an M2M communication network [6]. Since M2M devices are uplink-centric, it is thus important to design new resource allocation schemes specific to energy efficiency. In M2M networks, less traffic is generated on the downlink, therefore, providing solutions or more resources on the downlink is not solving the energy efficiency problem. Currently, to achieve energy efficiency for resource allocation on the uplink of M2M network, there may be certain degradation of other performances such as serving less M2M devices, inducing higher delay, including more signaling messages or limited QoS requirements, etc. Due to these issues, currently available solutions have left gaps.

**Figure 1:**
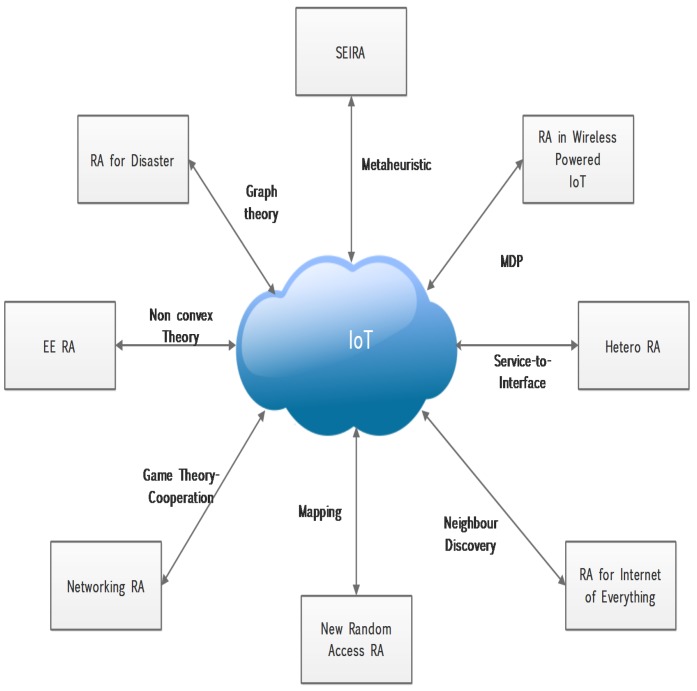
Popular recent resource allocation in IoT.

There is limited literature work for resource allocation in the MTC network to achieve energy efficiency; moreover, the existing techniques do not imply autonomous resource selection on the 5G network. Figure 1 shows the latest research regarding resource allocation in the IoT network with their related problem-solving approaches. The SEIRA [7] algorithm uses a metaheuristic technique for assigning resources to devices and claims to lower the communication cost. A resource allocation algorithm [8] is proposed for the wireless powered network. This algorithm implies the Markov Decision Process (MDP) and power allocation technique to maximize the throughput, whereas heterogeneous resource allocation [9] implements a service-to-interface to fulfill the service requirements while reducing the average cost of communication. A resource allocation technique [10] for the Internet of Everything introduces Energy Harvesting Active Networked Tags (EnHANTs) for managing power supply to devices deployed in cellular M2M network. A new random access technique [11] is proposed for resource allocation in a collision-free manner. It offers a scheduling protocol for the uplink channel. The literature [12] proposes a game theory that implies a cooperation strategy. An infrastructure-to-device (I2D) technique [13] is proposed that aims to maximize resource allocation for a device-to-device (D2D) network. The technique implements a weighted sum-rate (WSR) approach. In addition, the recent resource allocation algorithm [14] implements a maximum bipartite graph approach for assigning immediate resources during a disaster situation.

In summary, this paper has proposed an energy-efficient resource allocation threshold controlled access (TCA) scheme based on the device power profile using the 5G communication network. Our main concern is on small MTC devices such as health care sensors. In the first step, the device consumed power is compared with a selected maximum power budget value taken from the QoS metric based on IoT applications power requirements. Accordingly, the number of subcarriers is calculated. In the second step, the solution is optimized by including a threshold. In the next step, a suitable threshold value is selected. The threshold value enhances the calculation of subcarriers for less power consuming devices. The rest of the paper is organized as follows: Section 2 discusses previous related work followed by Section 3, which elaborates on the system model and problem formulation. The solution is proposed in Section 4. Simulation and results are discussed in Section 5, followed by Section 6, which presents the conclusions of the paper.

## 2. Related Work

As the interest grows in the M2M domain and in related IoT applications, more research initiatives have been raised worldwide including the energy efficiency topic. Previously, there has been a number of publications relating to achieving energy efficiency during resource allocation of M2M communication using the LTE network; Table 1 summarizes the work. Then, research work is discussed, and the literature work in [15] proposes a resource allocation algorithm for the contention-based access (CBA) method for MTC using an LTE-A network. By assessing the probabilities of events occurring in CBA methodology, transmission and latency are estimated according to CBA resource allocation. If latency is larger, increase the CBA resource allocation for a certain device, whereas Ref. [16] proposes a two-stage random access (RA) algorithm for allocating resources to user equipment. By using an access class barring (ACB) scheme proposed in the first stage, the user devices compete for resources according to the computed ACB factor. Later, in the second stage, the user devices that were ignored are served with new ACB check. The article [17] studied the usage of energy problem while using LTE-A uplink with Type 1 relays. The authors have proposed a heuristic methodology of spatial reuse of spectrum allocations and achieving less energy consumption by reducing the modulation and coding schemes (MCS) level. By using weights and reward calculation functions of game theory, the proper MCS levels can be selected, whereas the literature [18] introduces the quality of service (QoS) requirements’ constraint using a sum power minimization problem while achieving energy efficiency. The technique is based on QoS requirements of users and achieves the results through an inverse weed optimization algorithm. The results are simulated on LTE networks for co-existence of M2M/human-to-human(H2H). communication. In Ref. [19], the context information of a user device is used for scheduling purpose. The system is tested for the 5G network. Context information is based on LTE-A signaling architecture. A module based on context information is implemented in an LTE-A simulator that works on a priority scheduling algorithm.

In Ref. [20], the authors considered the uplink of the hybrid HetNet having femtocells overlaid on the macrocell, and is proposing a two-layer game-theoretic framework to achieve energy efficiency (EE) while increasing the usage of network resources. The proposed outer layer allows each femtocell access point (FAP) to maximize the use of the data rate for its users by opting for the frequency band ranging between sub-6 GHz and the mmWave. By applying the strategy of Nash equilibrium to this non-cooperative game, the proposed solution can be achieved, whereas the proposed inner layer ensures the energy efficiency to the user association method depending on the minimum data rate and maximum transmission power constraints for using a dual decomposition approach. The technique shows that the proposed hybrid HetNet scheme while using the mmWave frequency band improves the sum-rate and EE. Energy efficiency is also said to be achieved in [21], where the authors propose the device-to-device (D2D) communication architecture for utilizing the spectrum better. The energy-efficient resource allocation problem is considered a case study for the application of the mentioned sequential fractional programming algorithms. The main focus is on the downlink of an orthogonal frequency-division-multiple-access (OFDMA) cellular network coexisting with infrastructure-to-device (I2D) and D2D communications architecture. The paper presents the two key performance measures termed GEE and the weighted sum-rate (WSR) algorithms. Considering another paper [22], the authors propose a channel assignment algorithm. Using the forwarding weight of each node (LMFW) and the interference of local multicast. The proposed algorithm to improve the network performance considers partially overlapped channels and orthogonal channels. The simulation tends to show improvement in network allocation.

There still are many problems with currently available schemes in literature such as some of the algorithms requiring a big time delay and some strategies being complex to implement. There is a wide gap present for an effective resource allocation scheme specific for energy efficient M2M small devices—those are being ignored. Based on our knowledge, no one has investigated energy efficiency resource allocation for small powered devices on 5G coexisting with LTE-M and Narrowband-IoT networks. This network aims to be suitable for M2M communication; therefore, the proposed TCA algorithm is simulated on this network.

## 3. System Model and Problem Formulation

The MTC ecosystem usually consists of small devices, including sensors and actuators which are connected through the network. The network provides a communication means for sending and receiving data that is in a periodic form of M2M based communication. The cellular network has its popularity due to the large coverage and stability it provides [23]. MTC is expected to play a key role in the 5G network [24]. MTC devices are further divided into massive MTC (mMTC) and ultra-reliable MTC (uMTC) devices. We simulate the system in the 5G setting. In the system, our main concern is towards M2M low powered devices that have little power consumption.

5G introduces many waveforms including FBMC. It has gained much interest as a potential waveform in the 5G network. This technique is a pure physical layer concept. FBMC is a subclass of MC. FBMC modulation is considered in this system for an access technique in the 5G network [25]. In the case of the data link layer, 5G consists of Open Wireless Architecture, lower and upper network layers. With respect to resource blocks formation, they are distributed in both frequency and time domain. In the frequency domain, the resource blocks within available bandwidth are divided into carriers having 75 kHz spacing on the frequency bandwidth of 27 to 29 GHz.

### 3.1. System Description

A system model is based on the M2M devices mainly consisting of small sensors. The main focus of our research is related to biomedical sensors. The total number of available channels can be expressed as C, where C is the total number of channels. Within each channel, there are resource blocks RBs, which are expressed as RB[n]. Here, n represents the total count of subcarriers within each resource block. The reference signal power $P_{rsrp_{m,q}}$ [26] required by M2M device (*m*) while using resource blocks (*q*), during radio resource allocation can be computed as the below equation:

(1) $$P_{rsrp_{m,q}}=P_{BS}-10\log_{10}\left(q\times S_{q}\right).$$

Here, $P_{BS}$ is the power of the eNB node and $S_{q}$ is the number of subcarriers within each *q*th resource block. The total consumed power $P_{m,q}$ is further calculated by subtracting pathloss PL from reference power in Equation (2). The result is the estimated MTC device power. The purpose of this equation is to compute the general consuming signal power for a device to create the base calculations:

(2) $$P_{m,q}=P_{rsrp_{m,q}}-PL.$$

Signal-to-Noise Ratio (SNR) $\Theta_{m,q}$ [27] for the MTC device (*m*) while using resource blocks (*q*) is expressed as the following:

(3) $$\Theta_{m,q}=\frac{\left(P_{m,q}\times |h_{m,q}{|}^{2}\right)}{\delta^{2}}.$$

Here, in Equation (3), $h_{m,q}$ denotes channel fading amplitude, $\delta^{2}$ denotes power of Additive White Gaussian Noise (AWGN), and $P_{m,q}$ denotes consumed power by the M2M device (*m*). The data rate $D_{m}$ [28] that can be achieved by the following equation:

(4) $$D_{m}=B\times RB{\left[n\right]}_{m}\times \log_{2}\left(1+\Theta_{m,q}\right).$$

Here, *B* denotes effective bandwidth, and $RB{\left[n\right]}_{m}$ denotes the resource block assigned to a particular device. Power usage for all users can be measured as follows:

(5) $$P_{u}=\sum_{m=1}^{M}\sum_{k=1}^{RB}P_{m,k}.$$

The objective is to minimize the summed power $P_{u}$ while allocating appropriate resource blocks RB’s to MTC devices. The energy efficiency (EE) can be calculated through Equation (6). The units of energy efficiency are bits/joule:

(6) $$EE_{m}=\frac{D_{m}}{P_{m,q}}.$$

### 3.2. Channel Model

The specific channel model for the 5G network is [29]. RF coverage distances indicate path loss. According to Friis free space path loss, it is inversely proportional to the carrier frequency square. For deployment, environment path loss should be estimated. MTC devices and base station gains, RF bandwidth, coding, and modulation techniques determine cell coverage. Formula (7) is used for estimating the path loss

(7) $$\begin{array}{c} PL\left(l\right)=PL\left(l_{o}\right)+10\times k\times log_{10}\frac{l}{l_{0}}\\ +Y_{Rx}+Y_{C_{f}}+Y_{\theta }, \end{array}$$

where

(8) $$Pl\left(l_{o}\right)=20\times log_{10}\left(\frac{4\times \pi \times l_{o}}{\gamma }\right).$$

Here, in the equation, $PL(l_{o})$ represents the path loss at distance lo, $Y_{Rx}$ and $Y_{C_{f}}$ denote receiver heights and frequency correction factor, respectively, whereas $Y_{\theta }$ denotes standard deviation θ in dB of shadowing variable with mean 0 dB. The variable k denotes terrain models used in service areas.

### 3.3. Interference Model

**Figure 2:**
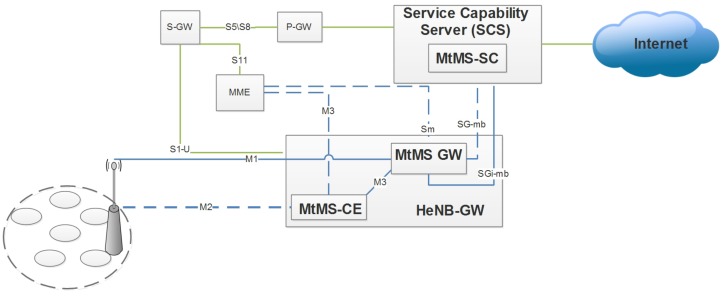
Interference reduction architecture.

For handling interference, we opt for machine-type multicast service model (MtMS [30]. MtMS defines the transmission procedures, related control with architecture for handling M2M communication multicast traffic, shown in Figure 2. MtMS serving center (MtMS-SC) initiates an MtMS session, which is implemented as part of SC and is the main source content of MtMS. For M2M devices, the anchor point is the service capability center (SCS), whose functionality is to send and receive data to and from the MTC devices. MtMS-SC is provided with parameters from SCS under the current MtMS session. MTC devices have the option of connecting with a suitable group of machines that are required to be served. After joining the group through the procedure, tracking area information is provided to a MtMS coordination entity (MtMS-CE) by MME. The tracking area information is relevant to devices that begin paging by using M3 control interfaces. MtMS-CE handles the joining procedure. After successful completion of the joining procedure, MtMS gateway (MtMS-GW) is responsible for data delivery in the current MtMS session through a balanced allocation of frequency resources with relevant transmission properties. In enhanced group paging, the MtMS devices are split into subgroups and paging is performed on the properties of subgroups. The size of the subgroup and paging time interval depends on the available resources on the current radio interface.

### 3.4. Transfer Rate Model

To estimate proper energy conservation, the data transfer rate should also be modeled. Since the paper targets a 5G network, it may therefore include cellular communication or D2D communication and any type of communication that may have different data rates. For this reason, we model the data transfer rate in the following Equation (9):

(9) $$TR_{c}=\sum_{k=1}^{n}\log_{2}\left[\frac{1+P_{c}\times G}{\sum_{i=1}^{m}P_{D}\times G+\theta^{2}}\right].$$

Here in the equation, $TR_{c}$ represents cellular data transfer rate, $P_{c}$ is transmission power required by cellular user data transfer, $P_{D}$ is transmission power required by D2D data transfer, *G* is the channel gain computed in Section 5 and θ is channel noise value. Similarly, the data transfer rate for D2D communication type can be computed as Equation (10):

(10) $$TR_{D}=\sum_{i=1}^{m}\sum_{k=1}^{n}\log_{2}\left(1+\frac{P_{D}\times G}{P_{c}+\sum_{i=1}^{m}P_{D}\times G+\theta^{2}}\right).$$

Here in the equation, $TR_{D}$ represents the D2D data transfer rate.

## 4. Energy Efficient Resource Allocation with Threshold Optimization

The main objective of our proposed TCA solution is to allocate resources according to the energy consumption of smaller devices. Since each device has different power profiles according to its related application, the QoS factor should also be included. Our proposed solution is in two-phases: phase A is an approach of resource allocation according to a QoS power profile metric and, in phase B, an optimal solution is provided through using a threshold for making the resource allocation enhanced especially for smaller devices. The mathematical notations used throughout the paper are listed in Table 2.

### 4.1. TCA Energy Efficient Resource Allocation

Each resource block is indexed as RB[n]. The received power of the device is compared with the set highest power limit of ⊘. Through Equation (11), power rank Γ is calculated:

(11) $$\Gamma =\left(1-\left(\frac{s(\varphi_{p})}{⊘}\right)\right).$$

Here, Γ is the calculated power rank through Equations (11) and (14). By using Γ value, the number of carriers are estimated that should be assigned to the device:

(12) $$\vartheta_{m}=\left(s(\varphi_{p}\right)\times \frac{1}{\Gamma })-PL,$$

(13) $$a=\frac{\left(\vartheta_{m}-P_{BS}\right)}{10},$$

(14) $$\lambda_{m}=10^{a}.$$

$\lambda_{m}$ is the estimated number of carriers in Equation (14) that will be assigned to the device. The proposed algorithm is shown in Algorithm 1. The achievable maximum data rate for each device on a subcarrier can be expressed as follows:

(15) $$b=10^{a},ifb<1b=100\times b;$$

(16) α=b×β.

**Algorithm 1** TCA algorithm
1:Initialize ⊘;2:Compute power rank Γ, 3:
$\Gamma =\left(1-\left(\frac{s(\varphi_{p})}{⊘}\right)\right)$
4:Calculate least power required $\vartheta_{m}$5:
$\vartheta_{m}=\left(s(\varphi_{p}\right)\times \frac{1}{\Gamma })-PL$
6:By using $\vartheta_{m}$ suitable carriers $\lambda_{m}$ are computed7:
$\vartheta_{m}=\left(s(\varphi_{p}\right)\times \frac{1}{\Gamma })-PL$
8:
$a=\frac{\left(\vartheta_{m}-P_{BS}\right)}{10}$
9:
$\lambda_{m}=10^{a}$ **return** $\lambda_{m}$



**Figure 3:**
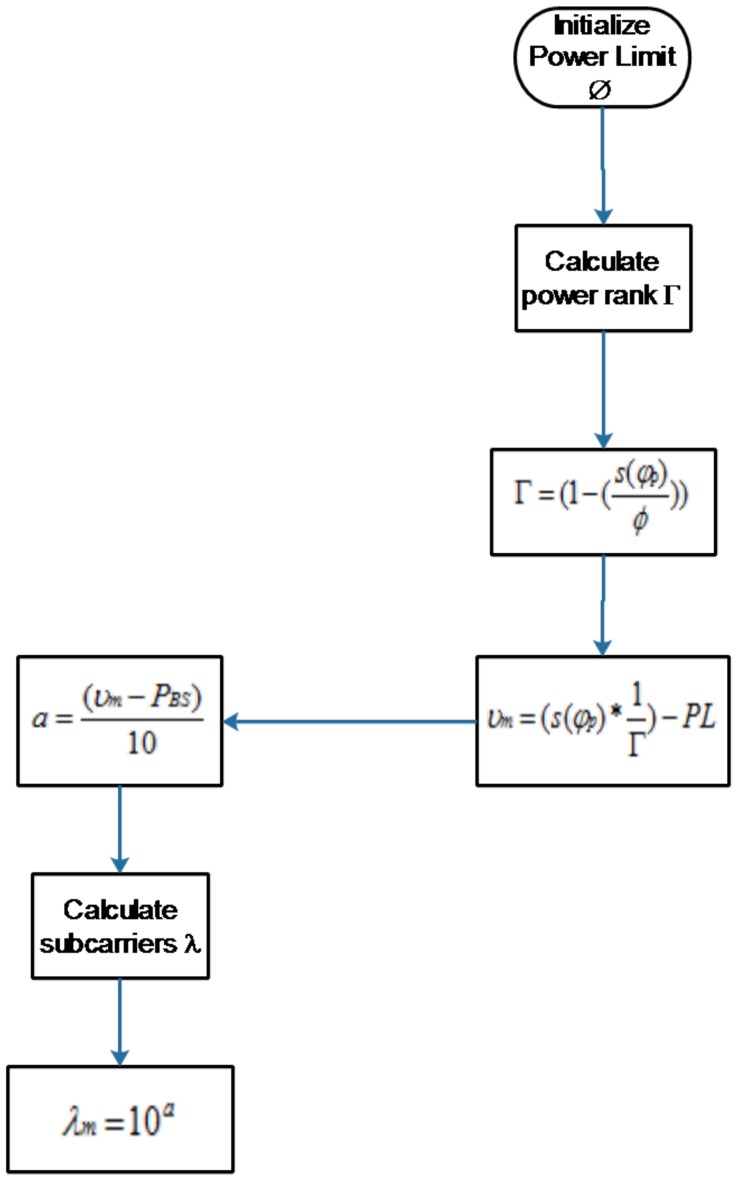
Flowchart of TCA algorithm.

Here, α is the computed number of carriers and β are the total number of available carriers. For simplification, the TCA algorithm is shown in flowchart Figure 3.

The value of the $\vartheta_{m}$ i-e the calculated power by estimation and the number of carriers $\lambda_{m}$ can be calculated from Equations (12)–(14), respectively.

### 4.2. QoS Power Metric

The above algorithm can be adjusted according to the requirement of the domain. Since each M2M application has different priority levels and relative device power profiles, a QoS metric listed in Table 3 is required for specifying the highest power limit ⊘ value used in Equation (11). The proposed metric is shown in Table 3.

Each resource block is pre-computed in groups/cluster according to the defined ranges of power they consume. A set of groups can be defined and resource blocks are assigned to each group according to power range, shown in Equation (14).

### 4.3. Controlled Threshold in the Algorithm

For proposing an optimal solution, we have introduced ω in Equation (17). ω is the threshold that updates the value of Γ used in Equation (12) for computing the number of carriers. By selecting a proper threshold, carriers’ allocation can be enhanced for especially small devices whose power usage is restricted; the algorithm is shown in Algorithm 2 by using the “interp1” interpolation function in Matlab 2017 version that creates a relationship between MTC device power and threshold. The function output according to QoS metric is shown in Table 3.

(17) Γ=ω×Γ.

**Algorithm 2** Optimization algorithm
1:Initialize QoS metric accordingly;2:Set required power limit points in the interpolation set.3:Interpolation function “interp1” select threshold “ω”4:ω enhances Γ value5:Γ value updates Equation (14).6:**return** Equation (14) gives no of carriers.


### 4.4. Complexity Analysis

In this section, the complexity of the proposed algorithm is analyzed. The whole algorithm is broken into two phases. In the proposed algorithm there are no comparisons, thus eliminating major computations that also require most of the time. In phase A, a simple constant computation is required that computes the percentage of device power relative to the set highest power limits. The complexity will be constant O(1). In the next step reference, power value and the assigned number of carriers are computed again in constant time. Therefore, the complexity of phase A does not exceed O(1). In phase B, a threshold is set that enhances the performance of M2M devices. A *n* number of comparisons are used according to the device power and QoS metric. Thus, the overall algorithm complexity falls in O(n) time.

### 4.5. Use Case Study

To deeply understand the effects and for the future implication of the proposed algorithm, certain effective use cases are discussed below, since low powered devices such as sensors’ networks are typically application-oriented silos that have difficulty in adjusting to high mobility and lack dynamic reconfigurability. 5G oriented SDN/NFV offers cost-effective solutions for improving the flexibility and agility of IoT networks. Our proposed algorithm gives priority to low powered devices for managing resource allocation within their QoS related highest power limits.

#### 4.5.1. Healthcare Applications

Use of low powered devices is increasing as these devices are low cost, have low latency and are crucial for collecting sensitive and periodic data related to patients. Such devices are also used to monitor sugar levels, heart rates, blood pressure and many other preserving clinical data. In the 5G network, these devices must survive to send periodic or chucked data in a continual manner. For this reason, the proposed algorithm assesses the highest power limits and adjusts the subcarriers’ allocation to each device differently and dynamically to achieve the desired throughput.

#### 4.5.2. Assets Tracking

It is predicted that the use of ultra low powered devices will be tripled by the year 2022 for use in asset tracking. Any kind of theft, damage or loss of any product chain is monitored by these devices. Such devices require emergency data communication, therefore, our proposed algorithm gives priority to the throughput of these devices.

## 5. Simulation Results and Discussion

The performance is shown and discussed in this section of our proposed algorithm. The simulation is performed in a Matlab simulator. The results of the simulation are presented for both service probability and energy efficiency. The throughput probability is the measurement of successful resource allocation to devices within the available bandwidth. It evaluates the proposed algorithm’s ability to provide a feasible solution under certain SNR.

### Simulation Results in 27 GHz

The algorithm is simulated on 27 GHz to 29 GHz bandwidth. The spacing between carriers is 75 kHz. For the channel, the path loss is set to 30 considering LOS conditions, 7 db is Rayleigh channel amplitude and −11 db set for Rayleigh signal power. The parameters values used are related to 5G system model [29]. The results of the proposed solution are compared with standard Round Robin (RR) and Best Channel Quality Indicator (BCQI) algorithms. RR allocates an equal number of resource blocks to devices, whereas BCQI gives priority to the device having the highest SNR value. It is a channel aware algorithm. For the testing scenario, different values of the threshold are used that are related to Table 3 values referring to device power profiles according to applications. We have partitioned the resource blocks to a set of subcarriers for better allocation and for a 5G network setting that can consist of any capillary networks. For evaluating the scenario, the following results are discussed.

**Figure 4:**
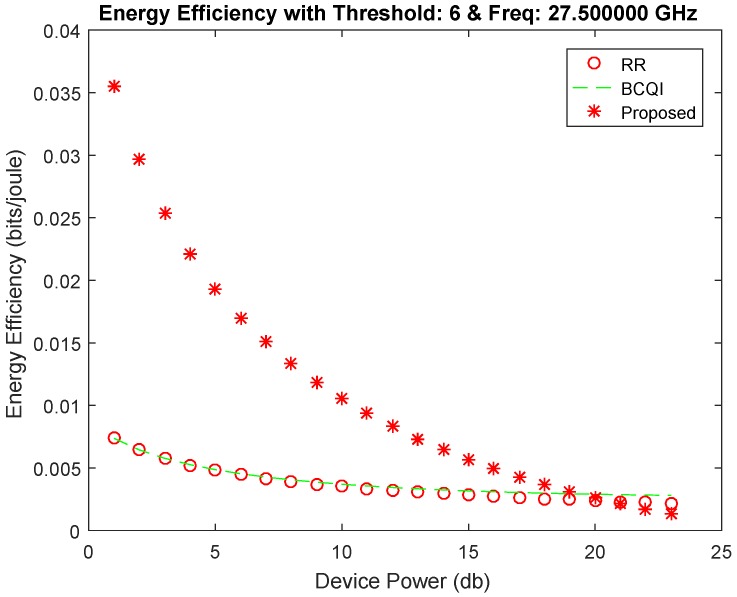
Energy efficiency achieved at threshold 6.

**Figure 5:**
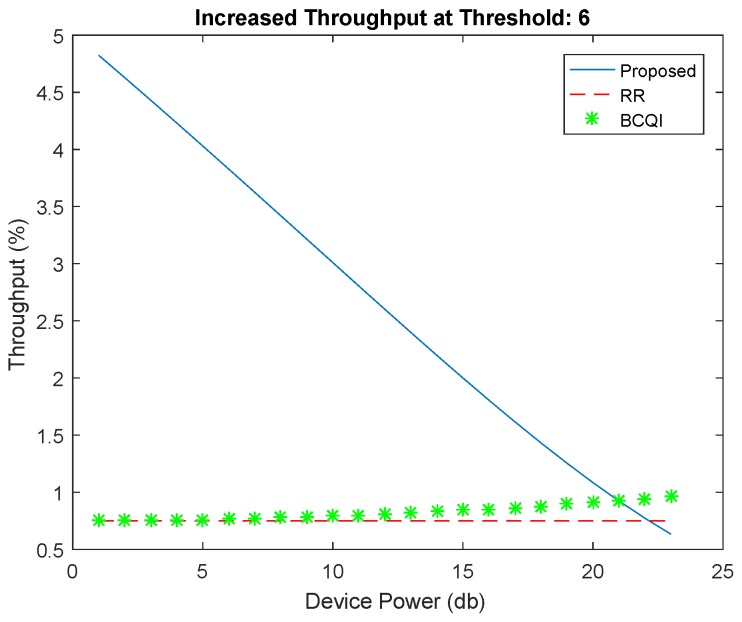
Throughput at threshold 6.

**Figure 6:**
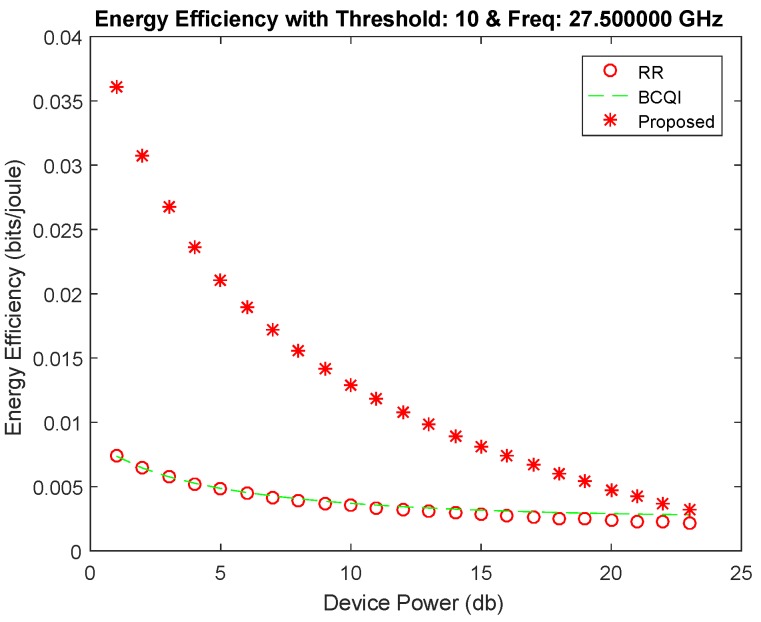
Energy efficiency achieved at threshold 10.

**Figure 7:**
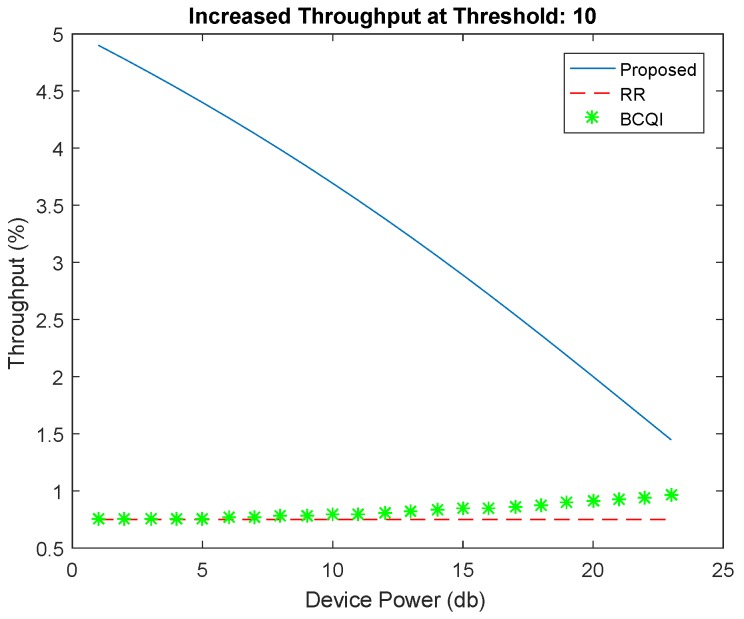
Throughput at threshold 10.

Figure 4 shows better performance of the proposed algorithm against RR and BCQI techniques, especially for M2M devices consuming less power consumption. The proposed solution achieves 30 percent better energy efficiency for small devices such as sensors used in different fields such as home automation, e-health, solar devices, etc. The highest power limit is set for 40 dB, which is the power usually used for small sensors mostly having the power supply through energy harvesting. Similarly, Figure 5 shows better throughput probability at threshold 6 of the proposed algorithm. It achieves more than 50 percent better resource allocation as compared with other techniques for low powered devices. The proposed solution is tested with different thresholds ranging from 6 to 15 further on the data rates that become limited. However, the throughput probability falls below RR and BCQI when the device power value increases, thereby requiring more power, which extends the power limit set to above 40 dB. Figure 6 illustrates better energy efficiency results at threshold 10. When the power level set to 40 dB, the achieved energy efficiency as shown reaches the highest attainable level that is 37% of energy efficiency. With comparison to RR and BCQI, it is more than 30%. Figure 7 illustrates the throughput probability plot achieved when the threshold level is set to 10. The threshold is increased to 6 dB which can tolerate increasing BER. However, RR and BCQI still give their throughput probability in increasing BER.

**Figure 8:**
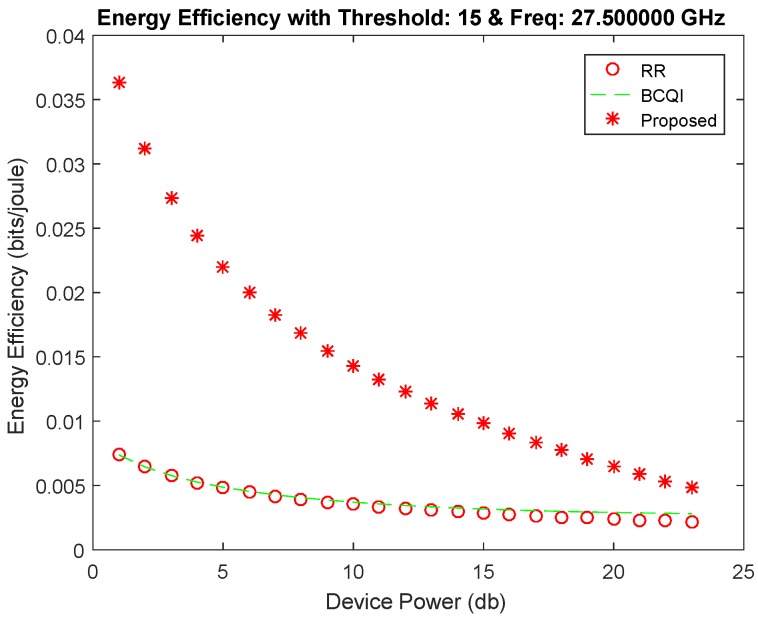
Energy efficiency achieved at threshold 15.

**Figure 9:**
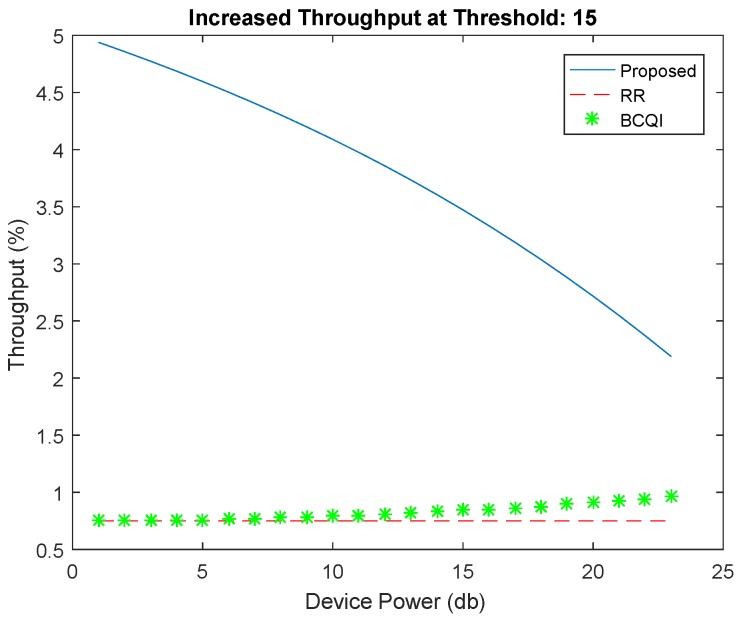
Throughput at threshold 15.

**Figure 10:**
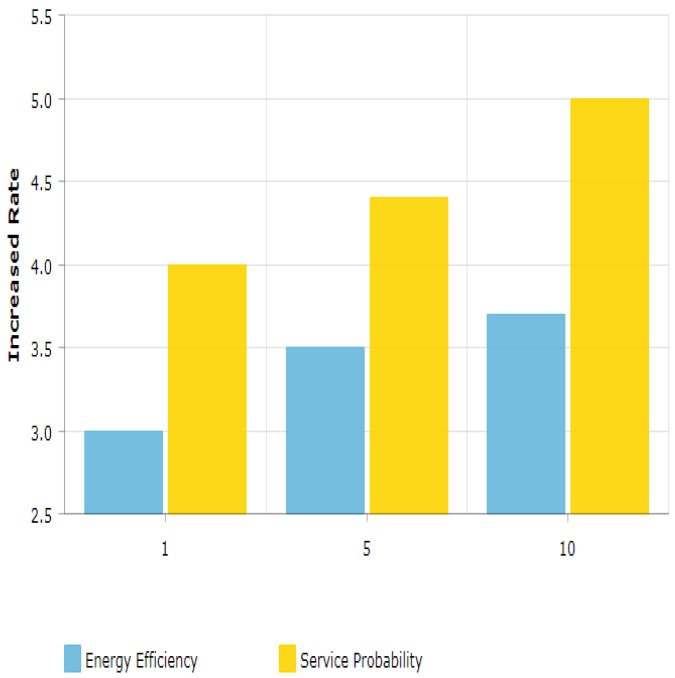
Comparison of results with different thresholds.

Figure 8 remains almost the same as in the case of threshold level 10, but it can also achieve energy efficiency for devices with increased device power. It can be observed that it has more achievable energy efficiency for high power devices. The highest achieved energy efficiency is 37%. Beyond threshold 6 dB to 15 dB, results do not increase the highest energy efficiency, but it does improve the performance with respect to high device powers. From the result, it shows a smooth throughput probability at threshold 15 shown in Figure 9. The plot keeps its curve even in increasing device power and shows better throughput probability with the comparison to RR and BCQI. Figure 10 illustrates the comparison between results with different thresholds. The thresholds refer to the maximum power limit set during simulation. The graph shows energy efficiency and service probability comparison having the range from 1, 5 and 10.

## 6. Conclusions

Energy efficiency is a major requirement in M2M communication ecosystems. In this paper, we have investigated the issue of resource allocation for small conserving M2M devices while using the 5G network. Our objective was to increase energy efficiency while increasing autonomous resource allocation to MTC devices. We proposed the algorithm that allocates resource blocks according to the QoS metric that relates the M2M device power limits. For the optimal solution, which enhances the results especially for small devices through the set of thresholds, the complexity of the proposed solution is low and produces less time delay. The solution gives better performance results when compared with standard algorithms. For future work, our research focus will be on intelligent data transmission for the uplink stream of IoT networks serving a massive number of devices. While considering the 5G network for IoT applications, both the MTC device and each gateway have finite resources, such as computation resource, power resource, and spectrum resource. Our investigation will be to optimize resource allocation while using intelligent strategies through machine learning techniques. 

## Figures and Tables

**Table 1:** Related work summary.

Algorithm	Complexity	Application	Energy Efficiency
Dynamic RA [15]	O(*n*^2^)	Real time	2%
Two-Stage RA [16]	O(*n*^2^)	Transport	3%
RA in Relay [17]	O(log n)	Relay networks	40%
H2H RA [18]	O(W*n^2^)	Communication	5%
Context RA [19]	O(n)	Scheduling	20%
Game theory RA [20]	O(W*n^2^)	HetNets	50%
D2D RA [21]	O(*n^m^*)	e-learning	45%
Mesh RA [22]	O(|V| + |E|)	Real time	4%

**Table 2:** Notations used in paper.

Symbol	Definition
$m_{i}$	M2M *i*th device
$P_{m,q}$	Consumed device power
$P_{u}$	Total summed power
$G_{i}$	channel gain
$N_{o}$	channel noise
$C_{i}$	shared channel available for *i*th MTC device
$M(\varphi_{p})$	Matrix of transmit powers from M2M devices
RB[n]	matrix of resource block
⊘	maximum power usage limit
$P_{rsrp_{m,q}}$	reference signal power
*q*	number of subcarriers within RB
$P_{BS}$	base station power
Pl	pathloss
$\Theta_{m,q}$	Signal-to-Noise Ratio (SNR)
$\delta^{2}$	power of Additive White Gaussian Noise (AWGN)
$h_{m,q}$	channel fading amplitude
$D_{m}$	data rate achieved by ith device
*B*	effective bandwidth
$\lambda_{i}$	calculated number of carriers for *i*th device
$\vartheta_{i}$	calculated power by estimation for *i*th device
α	computed number of carriers
β	total number of available carriers
ω	threshold
$J_{o}$	constant depends on antenna characteristic
δ	path loss constant
Ψ	Rayleigh random variable

**Table 3:** QoS power metric.

Domain	Highest Power Limit	Priority
Health care	0–8 db	High
Surveying	0–10 db	Low
Control	5–9 db	High
Enterprise	2–15 db	Medium
